# Impact of Psychological Resilience on the Fear of Pain and Activity Recovery in Postsurgical Patients: Observational Cohort Study

**DOI:** 10.2196/63556

**Published:** 2025-02-07

**Authors:** Yang Luo, Sisi Li, Lijuan Feng, Junyi Zheng, Chunfen Peng, Lihong Bao

**Affiliations:** 1 Department of Thoracic Surgery, Union Hospital, Tongji Medical College, Huazhong University of Science and Technology, 1277 Jiefang road, Jianghan District, Wuhan, 430022, China, 86 13886120866; 2 Department of Colorectal and Anal Surgery, Zhongnan Hospital of Wuhan University, Wuhan, China; 3 Cancer Center, Union Hospital, Tongji Medical College, Huazhong University of Science and Technology, Wuhan, China

**Keywords:** psychological resilience, fear of pain, postsurgical recovery, fear avoidance belief, pain management, activity recovery

## Abstract

**Background:**

The fear of pain (FOP) and fear avoidance belief (FAB) play a crucial role in the occurrence and development of chronic pain. However, the dynamics of these factors in postsurgical pain appear to differ, with the FOP often learned from others rather than directly caused by painful experiences. Psychological resilience refers to an individual’s capacity to adapt effectively to adversity, challenges, and threats, and may play a significant role in overcoming the FOP and avoidance behavior.

**Objective:**

The aim of this study was to investigate the role of psychological resilience in overcoming the FOP and avoidance behavior among surgical patients undergoing lung surgery.

**Methods:**

Participants were recruited at the Wuhan Union Hospital. Psychological resilience was measured using the Connor-Davidson Resilience Scale. The FOP was assessed using the simplified Chinese version of the Fear of Pain-9 items. The FAB was measured using the Physical Activity subscale of the Fear-Avoidance Beliefs Questionnaire. Activity recovery was assessed through questions related to social activities and household responsibilities. The adaptive least absolute shrinkage and selection operator (Lasso) regression analysis under nested cross-validation was used to identify key factors affecting postoperative FOP and activity recovery.

**Results:**

A total of 144 participants were included in the final analysis. The results showed that preoperative FOP (coefficient=8.620) and FAB (coefficient=8.560) were mainly positively correlated with postoperative FOP, while psychological resilience (coefficient=−5.822) and age (coefficient=−2.853) were negatively correlated with it. These average *R*^2^ of these models was 73% (SD 6%). Psychological resilience was the most important factor in predicting activity recovery, and these models obtained an average accuracy of 0.820 (SD 0.024) and an average area under the curve of 0.926 (SD 0.044).

**Conclusions:**

Psychological resilience was negatively associated with the postoperative FOP and positively with activity recovery in patients who underwent lung surgery. Patients with higher resilience are more likely to cope effectively with postsurgical pain and recover activities sooner. These findings highlight the importance of assessing and potentially enhancing psychological resilience in the perioperative period to improve postoperative outcomes.

## Introduction

Psychological factors such as the fear of pain (FOP) and fear avoidance belief (FAB) are thought to play an important role in the occurrence and development of chronic pain [1-3]. Based on the fear-avoidance model, among patients with chronic pain, painful experiences from activities will lead to the FOP, and then cause patients to refuse activities until disability, which in turn can lower the pain threshold and thus more easily lead to painful experiences after activities, forming a vicious cycle of fear reinforcement [4,5].

However, this seems to be different in the case of postsurgical pain. Our previous research [6] has shown that the FOP is related to postsurgical pain and can lead to avoidance behavior. A significant portion of this FOP is learned from others’ teaching or observational learning, while the FOP being directly caused by painful experiences is relatively rare. This seems to indicate that some people refuse to engage in certain activities such as coughing and ambulation from the beginning based on their cognition, rather than pain experience, as they have heard others tell them that such activities are dangerous.

A good pain experience after activities can help break the cycle of the FOP. The fear-avoidance model does not explain the dynamics underlying the FOP and functional recovery [4], such as how some postoperative patients can still continue to perform rehabilitation activities despite pain. It seems that there is an internal drive that prompts patients to overcome their fears and produce positive recovery behaviors.

Psychological resilience, also known as mental toughness [7], refers to an individual’s capacity to adapt effectively to adversity, challenges, and threats [8]. Psychological resilience has a positive impact in patients undergoing knee surgery and those with pain catastrophizing [9], as well as its predictive role in dental phobia [10]. However, its relationship with postsurgical pain and the FOP remains understudied.

Individuals with low psychological resilience tend to avoid stressful situations [11]. In this study, we aimed to understand the role of psychological resilience in overcoming the FOP and avoidance behavior among surgical patients. To this end, we followed up a sample from a previous study [12] to observe the impact of psychological resilience on the FOP and behavior, aiming to assist in postsurgical pain management.

## Methods

### Participants

This study was a single-center, cohort study of patients who were hospitalized to undergo thoracic surgery at the Wuhan Union Hospital, a tertiary hospital located in Wuhan, China, from May 2022 to January 2023. Participants were recruited based on the following inclusion criteria: (1) they were at least 18 years old; (2) they had undergone lung surgery; and (3) they did not have a history of neurological or psychiatric disorders. The exclusion criteria included individuals who experienced critical conditions during the study period or those who did not complete follow-up assessments. In the context where the sample size exceeds 10 events per variable [13], and considering a potential 10% dropout rate, we would terminate the further enrollment of participants.

### Ethical Considerations

This study was a follow-up study based on earlier research [12] (Registration number: ChiCTR2200056651), and has been approved by the institutional ethics board of Wuhan Union Hospital of Tongji Medical College, Huazhong University of Science and Technology (No. 20220026‐2). All the participants provided written and oral informed consent. The original informed consent form, which was approved by the same ethics committee, explicitly allows for secondary analysis without the need for additional consent (No. 20200351). Participants were provided with free counseling services during the follow-up period as a form of compensation. All study data have been anonymized or deidentified to protect the privacy and confidentiality of the participants.

### Fear of Pain

Before surgery and 6 months after surgery, we used the simplified Chinese version of the Fear of Pain-9 items (FOP-9) [14] to gather data on the FOP. The FOP-9 is a 5-point ordinal Likert scale where total scores range from 9 to 45, and a higher score signifies a greater level of FOP.

### Fear Avoidance Belief

On the third day after surgery, the Physical Activity subscale of the Fear-Avoidance Beliefs Questionnaire (FABQ-PA) was used to measure the FAB. The FABQ-PA is a self-reported questionnaire comprising the first 5 questions of the original Fear-Avoidance Beliefs Questionnaire [15]. A higher score on this subscale signifies a greater tendency towards the FAB.

### Psychological Resilience

Psychological resilience was collected 1 month after surgery by using the Connor-Davidson Resilience Scale (CD-RISC) [16]. The CD-RISC comprises 25 items, each rated on a 5-point scale (0‐4), with higher scores reflecting greater psychological resilience.

### Activity Recovery

Activity recovery was defined as the recovery of social activities and housework after discharge. Activity recovery was assessed at 6 months after surgery through questions such as, “Are your current social activities, like work and community activities, similar to what they were before the surgery?” and “Have your current family responsibilities returned to what they were before the surgery?” The answers were categorized as being lower or similar/higher than they were before surgery. Both the answers of “similar/higher” meant that the activity recovery was positive.

### Statistics

We used the adaptive least absolute shrinkage and selection operator (Lasso) with nested cross-validation to conduct variable selection, coefficient estimation, and performance prediction. All the statistics were completed using Python 3.12 (Python Software Foundation) and the scikit-learn library (developed by a community of open-source contributors, initially started by David Cournapeau). The process was as follows [17]: (1) Feature preprocessing: We conducted standardization on numeric features and one-hot encoding on categorical features. In order to avoid the problem of multicollinearity, we set the first one as the baseline and dropped it for each categorical variable, such as “Male” in sex, “Primary School” in education, “Presence” in chronic pain, and “Wedge Resection” in the surgery method. (2) Model training: We estimated the initial coefficients using Ridge regression with 5-fold cross-validation, and then conducted model training with the adaptive Lasso with 5-fold cross-validation after weighing the coefficients. (3) Model evaluation: In the 5-fold outer cross-validation, we selected the optimal model and calculated the average value of the model fitting parameters. (4) Variable estimation: We reported the regularization parameters and variable coefficients of the optimal model.

## Results

### Characteristics

**Figure 1.**
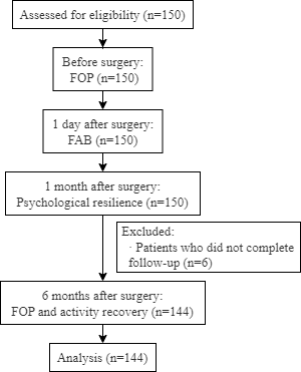
Flowchart of the research process and data collection. FOP: fear of pain; FAB: fear avoidance belief.

A total of 150 patients met the study’s inclusion criteria. However, 6 (4%) patients were excluded as they were lost to follow-up. A total of 144 participants were included in the final analysis, and the baseline characteristics are listed in Table 1. The study flow is shown in Figure 1.

**Table 1.** Baseline patient characteristics (n=144).

Characteristics	Value
Age (years), mean (SD)	52.40 (13.37)
Sex, n (%)	
Male	73 (50.7)
Female	71 (49.3)
Education, n (%)	
Primary school	20 (13.9)
Middle school	30 (20.8)
High school	43 (29.9)
University	51 (35.4)
Chronic pain, n (%)	
Absence	137 (95.1)
Presence	7 (4.9)
Preoperative FOP^a^, median (IQR)	16 (13-23)
Surgery method, n (%)	
Wedge resection	24 (16.7)
Segmental resection	64 (44.4)
Lobectomy	56 (38.9)
Pain intensity, median (IQR)	3 (1-4)
Fear avoidance belief, median (IQR)	11 (8-15)
Psychological resilience, mean (SD)	49.54 (12.99)
Activity recovery, n (%)	
Lower	38 (26.4)
Similar/Higher	106 (73.6)
Postoperative FOP, median (IQR)	20 (14-28)

a FOP: fear of pain.

### Postoperative FOP as the Dependent Variable

The statistical analysis showed that the mean squared error was 14.273 (SD 3.254), indicating a reasonable prediction error range with room for improvement. The mean *R*^²^ value was 0.731 (SD 0.060), suggesting that the models could account for approximately 73.1% (SD 6%) of the variance in postoperative FOP and had a relatively good fit. The optimal alpha of the optimal model was 0.081. The coefficient of the optimal model revealed that the preoperative FOP, FAB, and pain intensity had significant positive effects on the postoperative FOP with coefficients of 8.620, 8.560, and 0.417, respectively. In contrast, psychological resilience and age had significant negative impacts, with coefficients of −5.822 and −2.853, respectively. Among categorical variables, patients with a university education had lower postoperative FOP compared to those with primary school education, while female patients had a higher postoperative FOP than male patients did. The surgical method and chronic pain had no significant effect on the postoperative FOP (Table 2).

**Table 2.** Optimal model of adaptive least absolute shrinkage and selection operator (Lasso) regression for patients who underwent lung surgery in predicting the postoperative fear of pain (FOP; n=144).

Independent variables^a^	Coefficient
Age	–2.853
Sex	
Male	0.000
Female	0.778
Education	
Primary school	0.000
Middle school	−0.000
High school	0.000
University	−0.190
Chronic pain	
Absent	0.000
Present	0.000
Preoperative FOP	8.620
Surgery method	
Wedge resection	0.000
Segmental resection	-0.005
Lobectomy	0.000
Pain intensity	0.417
Fear avoidance belief	8.560
Psychological resilience	−5.822

a Lambda is 0.081 for all variables.

### Activity Recovery as the Dependent Variable

The model performed excellently after analysis, with an average accuracy of 0.820 (SD 0.024) and an average area under the curve of 0.926 (SD 0.044), indicating that the model had a good predictive ability and stability for activity recovery. Psychological resilience, FAB, and pain intensity were significant factors that affected the activity recovery, with coefficients of 1.185, −0.966, and −0.170, respectively. Both university education and segmental resection significantly positively affected activity recovery compared to baseline, with coefficients of 0.283 and 0.042, respectively. Age, sex, chronic pain, and preoperative FOP had no significant effect on activity recovery (Table 3).

**Table 3.** Optimal model of adaptive least absolute shrinkage and selection operator (Lasso) logistic regression for patients who underwent lung surgery in predicting activity recovery (n=144).

Independent variables^a^	Coefficient
Age	0.000
Sex	
Male	0.000
Female	0.000
Education	
Primary school	0.000
Middle school	0.000
High school	0.000
University	0.283
Chronic pain	
Absent	0.000
Present	0.000
Preoperative FOP^b^	0.000
Surgery method	
Wedge resection	0.000
Segmental resection	0.042
Lobectomy	0.000
Pain intensity	−0.170
Fear avoidance belief	−0.966
Psychological resilience	1.185

a Lambda is 2.783 for all variables.

b FOP: fear of pain.

## Discussion

### Study Findings

This study delved into the role of psychological resilience in perioperative FOP and avoidance behavior among surgical patients, particularly those undergoing lung surgery. The findings contribute novel insights to the existing literature, suggesting that psychological resilience may serve as a crucial factor in breaking the vicious cycle of FOP and facilitating recovery.

### Impact of Psychological Resilience on Postoperative FOP

Our study employed adaptive Lasso regression under nested cross-validation to analyze the factors influencing postoperative FOP, a common phenomenon experienced by patients following surgery [18]. The developed model demonstrated robust performance, with a high proportion of variance explained in postoperative FOP. Among the factors, psychological resilience emerged as the most significant inhibitory factor influencing postoperative FOP. Preconceived biases regarding postoperative activities and pain can lead to the development of the FOP [19,20]. We observed that some patients strictly adhered to the doctor’s orders despite potential pain, while others rejected and avoided them. This disparity can be attributed to the varying levels of psychological resilience among individuals. Resilient individuals are more likely to accept and face pain, actively adjust their pain concepts, and gradually expose themselves to pain stimuli [21,22]. For instance, they may perceive postoperative pain as a normal part of the recovery process and actively communicate with medical staff to learn pain management strategies, gradually adapting to and overcoming the FOP. Conversely, individuals with lower psychological resilience may avoid stressful situations, including postoperative activities, leading to a vicious cycle of pain, fear, and avoidance [9,10,23].

### Impact of Psychological Resilience on Postoperative Activity Recovery

The adaptive Lasso logistic regression exhibited higher accuracy and area under the curve values with a lower standard deviation, indicating that the model has high accuracy and stability in predicting postoperative activity recovery. The fact that the coefficient of psychological resilience is the largest revealed that psychological resilience had a stronger influence on postoperative activity recovery than other factors. This suggests that psychological resilience primarily enables individuals to initiate activities that may cause pain despite the presence of FOP, serving as a positive factor in overcoming fear. Patients with higher psychological resilience tend to have better ability to resume their activities postoperatively [24,25], and our research has also confirmed this. They may be more actively involved in social interactions and resume daily housework, thereby promoting physical function recovery and improving psychological well-being, resulting in a virtuous cycle. This may be because patients with high psychological resilience are better able to cope with negative emotions, have more reasonable cognitive expectations of the rehabilitation process, and can better overcome the fear of activities, thus promoting the recovery of postoperative activities [26,27].

### Study Limitations

This study has several limitations. First, psychological resilience was measured postoperatively, which may introduce bias due to the impact of surgery. Second, although both models demonstrated certain predictive abilities, there is room for improvement in the *R*^²^ values. Third, the generalizability of the findings is limited, as this study specifically focused on patients undergoing lung surgery, and the results may not be applicable to all surgical patients. Therefore, conducting multicenter studies with a bigger sample size would further validate the robustness of the results.

### Conclusion

This study revealed the significant role of psychological resilience in postsurgical pain management, particularly in reducing the FOP and promoting recovery of activities. Individuals with higher psychological resilience are more likely to effectively cope with postsurgical pain and reduce avoidance behaviors. These findings underscore the importance of assessing and potentially enhancing psychological resilience during the perioperative period, especially for those with higher levels of FOP. By fostering resilience and promoting correct pain cognition, clinical practitioners can empower patients to overcome the FOP, engage in rehabilitation activities, and achieve better postoperative outcomes.

## Abbreviations

CD-RISC: Connor-Davidson Resilience Scale
FAB: fear avoidance belief
FABQ-PA: Physical Activity subscale of the Fear-Avoidance Beliefs Questionnaire
FOP: fear of pain
FOP-9: Fear of Pain-9 items
Lasso: least absolute shrinkage and selection operator
